# Symptomatic and Subclinical Infection with Rotavirus P[8]G9, Rural Ecuador

**DOI:** 10.3201/eid1304.061285

**Published:** 2007-04

**Authors:** Pablo Endara, Gabriel Trueba, Owen D. Solberg, Sarah J. Bates, Karina Ponce, William Cevallos, Jelle Matthijnssens, Joseph N.S. Eisenberg

**Affiliations:** * Universidad San Francisco de Quito, Quito, Ecuador; † University of California, Berkeley, California, USA; ‡ University of Leuven, Leuven, Belgium; § University of Michigan, Ann Arbor, Michigan, USA

**Keywords:** Rotavirus, genotype analysis, Ecuador, epidemiology, community study, research

## Abstract

During the past decade, rotavirus genotype G9 has spread throughout the world, adding to and sometimes supplanting the common genotypes G1–G4. We report evidence of this spread in a population sample within rural Ecuador. A total of 1,656 stool samples were collected from both patients with diarrhea and asymptomatic residents in 22 remote communities in northwestern Ecuador from August 2003 through February 2006. Rotavirus was detected in 23.4% of case-patients and 3.2% of controls. From these 136 rotavirus-positive samples, a subset of 47 were genotyped; 72% were of genotype G9, and 62% were genotype P[8]G9. As a comparison, 29 rotavirus-positive stool samples were collected from a hospital in Quito during March 2006 and genotyped; 86% were of genotype P[8]G9. Few countries have reported P[8]G9 rotavirus detection rates as high as those of the current study. This growing prevalence may require changes to current vaccination programs to include coverage for this genotype.

## Abstract 2

Prevalence of this genotype is increasing.

Rotavirus is the most important cause of acute gastroenteritis and death in infants and young children worldwide, causing an estimated 352,000–592,000 deaths in children <5 years of age (*1*). Although the incidence of infection in children in industrialized and developing countries is similar, outcomes vary widely. In countries classified by the World Bank as high-income, the risk of dying from rotavirus before age 5 is 1 in 48,680; the equivalent risk in low-income countries is 1 in 205 (*1*).

The rotavirus genome is made up of 11 double-stranded RNA segments; each segment encodes a unique structural or nonstructural protein. A 3-layered protein coat encloses the genetic material. The VP2 proteins form the innermost layer, which is in turn surrounded by a sheet of VP6 proteins. The outer layer consists of 2 antigenic proteins, VP7 and VP4, also referred to as the G (glycoprotein) and P (protease-sensitive) proteins, respectively (*2**,**3*). To date, 15 G-protein genotypes and 24 P-protein genotypes have been identified (*3*), of which 10 G and 12 P types are known to infect humans. The combination of these 2 proteins constitutes the viral genotype (*4*). Because of the segmented nature of the rotavirus genome, the genes for the external structural proteins may segregate independently during coinfection (genetic reassortment), thus increasing the genetic diversity of rotaviruses.

Data from 1994 through 2003 indicate that the 4 most prevalent human rotavirus genotypes worldwide were P[8]G1 (52%), P[4]G2 (11%), P[8]G4 (8%), and P[8]G3 (3%), which together represented ≈74% of the global isolates (*2*). In Latin America during the same period, the prevalence of these 4 viral types was similar (*3**,**5*).

More recent data suggest that the G9 genotype has gained global importance during the past 10 years (3,6*–**9*). The P[8]G9 type, the most common combination, may have resulted from a reassortment event between the most prevalent type P[8] and a strain carrying G9. From 1990 through 2004, P[8]G9 rotaviruses caused <5% of rotavirus infections worldwide but 15% of infections in South America (*2**,**3*). A less common reassortment is P[6]G9 (*2**,**6*).

Genotyping of circulating strains has epidemiologic importance and relevance for vaccination planning. The most efficacious vaccination protocols are those that use viral serotypes similar to those circulating in a given community (homotypic responses) (*3*). Vaccines with serotypes distinct from those circulating (heterotypic) are less effective (*9**,**10*).

Although rotavirus infections have been reported in Ecuador (*11*), to our knowledge, this is the first report of circulating genotypes. The data presented here are unique in that they are community-based and include all symptomatic community residents as well as asymptomatic controls. This approach differs from most rotavirus genotyping studies, which focus on patients in a clinical setting. The data thus document the total illness rate associated with rotavirus infection from 22 remote, rural communities on the northern coast of Ecuador.

## Methods

### Study Population and Design

As part of a larger community-based case-control study, fecal samples were collected from persons in 22 remote communities located in Esmeraldas, the northernmost province on the coast of Ecuador. Each of 21 small, rural communities was visited 4 times, each time for 15 days, from August 2003 through February 2006. Fecal samples were also collected from the region’s largest town, Borbón, for 15 days in July 2005. During each 15-day visit, health workers visited every household and interviewed residents to identify every case of diarrhea. For each identified case of diarrhea, 3 asymptomatic controls were selected, 1 from the case-patient’s household and 2 randomly selected from the community. A total of 1,656 stools samples were collected, 411 (25%) from patients with diarrhea.

To determine whether the results in these remote communities were representative of rotavirus infections in other Ecuadorian locations, 29 fecal samples from children <5 years of age with rotavirus-associated diarrhea rotavirus were collected at the Hospital de Niños Baca Ortiz in Quito, which is an urban environment ≈200 km from the study area. Protocols were approved by the bioethics committee at the Universidad San Francisco de Quito and the Internal Review Board at the University of California, Berkeley, California, USA.

### Rotavirus Detection and Testing

All of the 1,656 samples (symptomatic and nonsymptomatic) were analyzed for the presence of rotavirus with a commercial immunochromatographic test (RIDA Quick Rotavirus, R-Biopharm AG, Darmstadt, Germany). All rotavirus-positive samples collected from February 2005 through February 2006 (n = 47) were preserved in liquid nitrogen and transported to Quito for PCR genotyping. The double-stranded rotavirus RNA was extracted from the stool specimens by using TRIZOL Reagent (Invitrogen Corp., Carlsbad, CA, USA) or the UltraClean Tissue RNA Kit (MoBio Laboratories, Inc., Carlsbad, CA, USA) according to manufacturer’s instructions. RNA was stored at –80°C until further use. A 2-step, seminested multiplex reverse transcription–PCR was carried out for G- and P-genotyping based on a protocol provided by the US Centers for Disease Control and Prevention (J. Gentsch, pers. comm.). Briefly, primers 9con1 and 9con2 were used for the first amplification of the VP7 gene and primers 9T-1, 9T-2, 9T-3P, 9T-4, and 9T-9B were then used to ascertain the G genotype (*12*). Primers Con3 and Con2 were used for the partial amplification of the VP4 gene and primers 1T-1, 2T-1, 3T-1, 4T-1, 5T-1, and ND2 were then used to ascertain the P genotype (*13*).

Viral RNA was denatured for 5 min at 97°C. Retrotranscription and the first amplification were carried out by using a SuperScript III RT/Platinum Taq polymerase kit (Invitrogen Corp.). Primers were used at 200 nmol/L each, and the 1× buffer provided by the manufacturer contained 1.6 mmol/L MgSO_4_ and 200 μmol/L of each deoxynucleotide triphosphate. The retrotranscription was carried out at 42°C for 45 min and stopped at 96°C for 2 min. The first amplification consisted of 30 cycles at 94°C for 30 s, 50°C for 30 s, and 72°C for 60 s. The second amplification was carried out by using PuReTaq Ready-To-Go PCR beads (Amersham Biosciences, Piscataway, NJ, USA) and primers at a final concentration of 400 nmol/L. The cycling parameters were 30 cycles at 94°C for 30 s, 42°C for 30 s and 72°C for 60 s, and a final extension at 72°C for 1 min. Electrophoresis of the PCR product was conducted on 1.8% agarose gels at 60 volts and visualized under ultraviolet light.

### Nucleotide Sequencing

For sequencing purposes, samples were transferred directly onto chromatography paper strips treated with sodium dodecyl sulfate–EDTA, dried overnight at room temperature, and sent to Belgium by standard postal service (*14*). From the community samples, 22 PCR products that were identified as P[8]G9 were purified with the QIAquick PCR purification kit (QIAGEN, Hilden, Germany), and sequenced with the ABI PRISM BigDye Terminator Cycle sequencing reaction kit (Applied Biosystems, Foster City, CA, USA) on an ABI PRISM 3100 automated sequencer (Applied Biosystems). Primers Beg9 and End9 were used for the VP7 gene (*15*) and primers 1-17F (*16*) and Con2 were used for the VP4 gene. The sequencing reaction conditions were 25 cycles at 94°C for 15 s, 50°C for 15 s, and 72°C for 4 min, and a final extension of 72°C for 7 min.

### Sequence Analysis

Partial VP7 DNA sequences from 22 G9 community samples and 17 additional G9 VP7 sequences obtained from GenBank for comparison purposes were aligned by using ClustalW (*17*). A phylogenetic tree was constructed by using a maximum likelihood algorithm as implemented by DNAML in PHYLIP (*18*). A VP7 sequence from a G3 genotype rotavirus was used to root the tree. Bootstrap support was calculated by using 500 bootstrapped data replicates as implemented by SEQBOOT in PHYLIP. VP7 gene sequences from the 22 G9 community isolates were deposited in GenBank under accession nos. DQ848566–DQ848587.

## Results

Of 1,656 fecal samples from remote communities analyzed for rotavirus, 136 (8.2%) were determined to be positive by the commercial immunochromatographic test. Of these positive samples, 96 were from the 411 patients with diarrhea and 40 were from the 1,245 asymptomatic controls. Diarrhea was significantly associated with being infected with rotavirus (odds ratio = 9.2; 95% confidence interval 6.1–13.9). Rotavirus RNA was detected at the highest rates from symptomatic infants and, surprisingly, persons >40 years of age (Table 1). No pronounced seasonality of rotavirus infection was determined, and incidence was not significantly associated with month of collection or with the observed 30-day rainfall for 15 days before the visit and during the visit (data not shown). This lack of seasonality in the tropics has been reported previously (*19*).

**Table 1:** Age distribution of 411 case-patients (those with diarrhea) and 1,245 controls (those without diarrhea) from rural communities of Esmeraldas, Ecuador*

	Age group, y					
<1	1–<5	5–<20	20–<40	>40	Total
Case-patiants +/N (%)	20/69 (29.0)	33/181(18.2)	16/64 (25.0)	0/17 (0)	11/34 (32.4)	96/411 (23.4)
Controls +/N (%)	0/35 (0)	6/142 (4.2)	17/493 (3.4)	13/282 (4.6)	4/251 (1.6)	40/1245 (3.2)
OR (95% CI)	– (3.6–∞)	5.1 (2.0–15.2)	9.3 (4.1–20.9)	– (0–4.8)	29.5 (7.8–133.9)	9.2 (6.1–13.9)

*Samples collected Aug 2003–Feb 2006. + indicates number of positive results on immunochormatographic test. OR, odds ratio; CI, confidence interval. Confidence interval bands for the <1 y and 20–<40 y age groups were obtained by using the Fisher exact test.

From the 136 rotavirus-positive community samples, a subset of 47 samples were genotyped for the VP4 and VP7 genes. This subset represented all samples collected from February 2005 through February 2006, from 14 rural communities and Borbón. Of these 47 samples, 35 (74%) yielded successful PCR typing results for the VP4 gene and 37 (79%) yielded successful PCR typing results for the VP7 gene. An additional 6 (13%) yielded successful PCR amplification at 1 of the 2 genes. The remaining 8 (17%) samples were not typeable. Six of these untypeable RNA samples, along with 6 typeable samples, were subjected to electrophoresis on an agarose gel and visualized by staining with ethidium bromide in an attempt to detect rotavirus RNA. None of the untypeable samples produced rotavirus RNA banding patterns, whereas 3 of 6 typeable samples could be visualized.

Among the successfully typed samples, genotypes P[8] and G9 predominated. A small proportion of the samples produced patterns corresponding to P[6]G1 and P[6]G9 (Table 2). Table 3 summarizes the percentage of patients infected with P[8], G9, and P[8]G9 based on 2 assumptions of the 13 samples in which 1 or both of the VP4 and VP7 genes were not typeable. The first assumption was that samples were nontypeable because they were degraded sometime between testing positive by immunochromatographic tests in the field and the sample’s arrival in the laboratory in Quito. In this case, we assumed that those samples were missing data. The second assumption was that the samples were nontypeable because they were novel strains, and we therefore included them in the dataset. G9 genotype was identified in 34 samples, resulting in a 72%–92% infection rate; P[8] genotype was identified in 31 samples, a 66%–89% infection rate; and the combination of P[8]G9 was found in 29 samples, a 62%–88% infection rate.

**Table 2:** Distribution of G and P types in rural communities of Esmeraldas and in Quito, Ecuador*

Location	P[6]G1	P[6]G9	P[8]G9	P[8]G_NT_	P_NT_G9	P_NT_G_NT_	P[4]/P[8] G2	P[4]/P[8] G2/G9	P[8] G2/G9	P[6]/P[8] G9	Total
Esmeraldas	3	1	29	2	4	8	0	0	0	0	47
Quito	1	0	21	0	1	1	1	2	1	1	29

*As determined by genotype specific, multiplex reverse transcription–PCR. Samples were collected from 14 rural communities and Borbón and from Quito, Feb 2005–Feb 2006. Undetermined types are designated NT. Coinfection with different genotypes is designated with a slash.

**Table 3:** Estimated percentages of rotavirus-positive persons infected with P[8], G9, and P[8]G9, Ecuador

Location	P[8], n (%)	G9, n (%)	P[8]G9, n (%)
Esmeraldas	31 (66–89)	34 (72–92)	29 (62–88)
Quito	26 (90–96)	26 (90–96)	25 (86–93)

*Assumes that all nontypeable samples were either degraded (upper bound) or a novel strain (lower bound).

To determine whether the genotypes in remote communities corresponded to strains circulating elsewhere in Ecuador, we analyzed 29 rotavirus-positive samples from Hospital de Niños Baca Ortiz in Quito. Again, genotypes P[8] and G9 overwhelmingly predominated (Table 2). Electrophoretic evidence for 5 mixed infections was found among these urban samples, but this was not seen among the rural samples (Table 2).

Of the 29 P[8]G9 community samples, 22 were sent for sequencing to the University of Leuven in Belgium. Approximately 750 bp of high-quality nucleotide sequence data for the VP7 gene was obtained from each sample. The 22 P[8]G9 samples were remarkably homogenous at the sequence level, with only 3 single nucleotide polymorphisms found in the 22 sequences. Phylogenetic analysis of the sequences (Figure) showed that the Ecuadorian sequences grouped together monophyletically and were part of the large clade composed of most of the recently isolated G9 rotavirus sequences worldwide.

**Figure:**
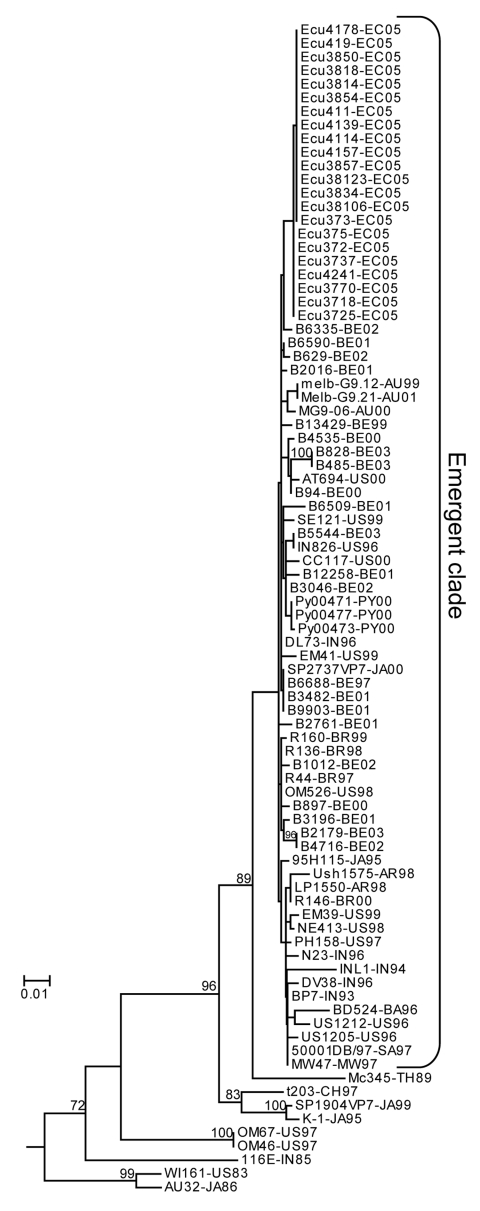
Maximum likelihood phylogenetic tree constructed from VP7 nucleotide sequences of G9 genotype rotavirus isolates. Taxa included are the 22 sequences from the current study and 65 sequences obtained from GenBank that represent global G9 rotavirus diversity. Taxa labels indicate isolate name followed by the country and year of collection. Country abbreviations: AU, Australia; BA, Bangladesh; BE, Belgium; BR, Brazil; CH, China; EC, Ecuador; IN, India; JA, Japan; MW, Malawi; PY, Paraguay; SA, Republic of South Africa; TH, Thailand; US, United States. GenBank accession nos. for the diversity isolates: SP2737VP7 (AB091752), MG9-06 (AY307085), Melb-G9.21 (AY307090), Melb-G9.12 (AY307088), EM41 (AJ491170), DL73 (AJ491165), Se121 (AJ491192), CC117 (AJ491153), In826 (AJ491173), At694 (AJ491159), 95H115 (AB045373), Ph158 (AJ491183), BD524 (AJ250543), INL1 (AJ250277), US1205 (AF060487), US1212 (AJ250272), MW47 (AJ250544), 50001DB (AF529864), N23 (AJ491177), BP7 (AJ491161), R146 (AF274970), DV38 (AJ491168), NE413 (AJ491178), EM39 (AJ491169), R136 (AF438228), Om526 (AJ491182), R44 (AF438227), R160 (AF274971), MC345 (D38055), T203 (AY003871), K1 (AB045374), SP1904VP7 (AB091754), Om46 (AJ491181), Om67 (AJ491179), AU32 (AB045372), 116E (L14072), WI61 (AB180969), Ush1575 (AF323711), LP1550 (AF323717), Py00471 (DQ015691), Py00473 (DQ015692), Py00477 (DQ015693). The 22 Belgian isolates were selected from the GenBank PopSet AY487853–AY487895. Bootstrap values >70 are shown on internal branches. The tree was rooted with the VP7 sequence of a G3 genotype rotavirus (AY740736).

## Discussion

The present study reports a high rate of infection (72%–96%) with rotavirus G9 genotype among persons in 2 geographically distinct regions within Ecuador, a remote coastal rain forest and an urban Andean hospital. To our knowledge, it is the first description of rotavirus genotypes in Ecuador, and the results support the observation that the G9 genotype, particularly P[8]G9, is spreading throughout Latin America. Also, the present study appears to be one of the few community-based descriptions of rotavirus infection (*20**–**22*). Symptomatic persons were actively identified in the community, recruited into the study, and matched with 3 asymptomatic controls each. This approach presents a more complete picture of rotavirus infection in rural communities than would be possible with the clinical sampling used in most previous studies that presumably focused on more urban environments. The high rate of rotavirus infection among symptomatic persons >40 years of age may be due to this age group’s lack of exposure to the emerging rotavirus genotype and is an observation that might have been missed in a purely clinical study.

The G9 genotype has been documented since the early 1980s (*23**,**24*). Throughout much of the 1980s and 1990s, G9 was considered very rare; however, recent reports have described it as increasingly important (*2**,**3**,**25*). In the United States, the G9 genotype was detected in a 1995–1996 outbreak (*8*) and maintained its presence in the subsequent 2 years, with an average detection rate across 10 US cities of 7% (*26*). In Australia, the overall G9 detection rate, averaged across 3 population centers, increased from <1% in 1997 to 29% in 2001 (*27*). In Japan, G9 was essentially undetected throughout the mid-1980s and 1990s until it suddenly appeared in several cities in 1998–1999 (*28*). In India, G9 strains were detected for the first time in the late 1980s and throughout the early 1990s were usually found in combination with the P[11] or P[6] genotypes at a detection rate of about 20% (*12*). A study of 6 population centers across India during 1996–1998 found an overall G9 detection rate of 17% but found G9 as the major strain (and for the first time associated mainly with P[8] genotype) in New Delhi in late 1998 (*29*). At 17 sites throughout the African continent during 1996–1999, the G9 detection rate was generally ≤5% (*30*), with the exception of Ghana (1997–1999), where it comprised 28% of rotavirus positive samples, and Nigeria (1998–1999), where it comprised 37% (*31*). In Europe, many instances of G9 detection have been reported from the late 1990s through the early 2000s (*16**,**32**–**34*).

Latin America, in particular, has seen a surge in dominance of this genotype in recent years. In Rio de Janeiro, Brazil, during 1997–1999, the detection rate was ≈15% (*6**,**7*). In São Paulo, Brazil, during 1996–2003, the rate was 17% overall but in the last 2 years, G9 accounted for 30%–50% of rotavirus infections (*35*). To our knowledge, only 4 studies have reported G9 detection rates as high as those in our study: 75% in Paraguay, 2000 (*36*); 75%–90% in Salvador, Brazil, 1999–2002 (*9*); 92% in Chiang Mai, Thailand, 2000–2001 (*37*); and 73% in Alice Springs, Australia, 2001 (*27*).

A potential source of bias in this study comes from the incomplete typing of 13% of the putatively rotavirus-positive specimens and the inability to type an additional 17%. These incomplete or untypeable samples, which were positive by immunochromatographic tests, may be the result of inappropriate handling or storage of some fecal samples, which can be complicated in remote community studies such as this. Typing failure because of sample degradation or false-positive ELISA results is not likely to result in biased results, and the lack of visualizable rotavirus RNA bands among the untypeable samples suggests that degradation is a likely cause of typing failure. However, in any PCR-based typing scheme, typing failure may be caused by primer–template mismatch, which could bias results, especially with novel strains (*16**,**38*). A nonsystematic review of 16 recent studies suggests that failure of PCR-based G-typing is relatively widespread, although the failure rate varies. Four studies report <5% typing failure (*16**,**33**,**35**,**37*), 3 report 5%–10% failure (*9**,**12**,**26*), 2 report 10%–20% failure (*29**,**36*), 2 report 20%–30% failure (*27**,**30*), and 2 report >30% failure (*31**,**34*). An additional 3 studies do not explicitly state whether all typed samples yielded results (*6**,**7**,**32*). A more complete picture of global rotavirus diversity should facilitate efforts to improve molecular typing techniques. In this study, the maximum possible bias would affect the Esmeraldas community results by lowering the G9 infection rate from 92% to 72% and the P[8]G9 from 88% to 62%. In the Quito samples the effect is much less pronounced, potentially lowering the G9 infection rate from 96% to 90% and P[8]G9 rate from 93% to 86%. Further studies are required to narrow this uncertainty; however, even the lower end estimates indicate that P[8]G9 is the predominant strain in Ecuador.

Additional evidence that G9 rotavirus is spreading through Latin America comes from comparing our nucleotide sequences to other sequences reported in GenBank. The sequences from the current study cluster into a large clade, which includes most of the recently isolated G9 rotavirus reported in the literature. This “emerging clade” is relatively homogenous: most isolates within the clade have <1% sequence divergence, an observation about G9 that has been made previously (*39*). However, the more recent regional isolates do tend to cluster together, as is the case for subclades composed of strains from Australia, Paraguay, or Ecuador (Figure). The low bootstrap support for these subclades is due to the small number of single nucleotide polymorphisms differentiating them.

The increasing prevalence of G9 rotavirus is particularly relevant given that many countries, including Ecuador, have approved the use of 2 rotavirus vaccines (*10**,**40*), but despite the wide distribution of G9 during the past 9 years (*2**,**3**,**6**–**9*), neither vaccine formulation includes the serotype G9 antigen (*10**,**40*). Studies have shown that some vaccines that do not contain the G9 antigen may still be capable of eliciting protective immunity against the G9 serotype (*10*). This immunity is most likely attributable to the G9 genotype’s common association with P[8], which is included in both vaccines. However, cross-immunity may not be universal, as has been seen with type P[4]G2 (*2**,**3**,**10*). Continual surveillance of circulating types, therefore, should be carried out before to the introduction and during the implementation of rotavirus vaccination programs.
